# Operation in the Treatment of Impervious Urachus1Read before the annual meeting of the Iowa and Nebraska Veterinary Medical Association, November 20, 1900.

**Published:** 1901-07

**Authors:** J. H. Gain

**Affiliations:** Seward, Neb.


### OPERATION IN THE TREATMENT OF IMPERVIOUS URACHUS.[1]

By J. H. Gain, M.D.C.,

SEWARD, NEB.

Until the later treatment for parturient apoplexy, the country practitioner had a chill when the symptoms of this trouble were described along with a request for help, and somewhat the same feeling comes over him when a stock-owner comes in and says that his young colt is unable to stand, passes urine in considerable quantities through the navel, has hocks badly swollen, and says the old mare stepped on them. During the past spring and summer we had a great number of these cases. A few that had only a slight discharge from the navel, with no other symptoms, recovered both with and without treatment. In the more severe cases, having tried various injections, together with tying off the urachus and loosening all of them, the following operation was suggested and performed by Dr. Anderson :

The patient, having been kept from dam for six hours, was laid down and tied so as to leave the abdomen freely exposed. An

[1] Read before the annual meeting of the Iowa and Nebraska Veterinary Medical Association, November 20, 1900.

anæsthetic was administered. The abdomen was thoroughly washed with soap and water, and the hair shaved from a space four inches wide by eight long with the navel as the centre. An elliptical shaped incision, five inches long and just wide enough to take in the navel, was made through the skin, underlying tissues, and the peritoneum. The umbilical vein was traced ahead until found to be healthy, ligated with silkworm-gut, and severed. The urachus was then followed up to the bladder and two strands of the silkworm-gut passed between the branches and each ligated separately, and a strand then passed around and over both ligatures. The urachus was then severed about a half-inch below the ligature. The peritoneum was closed with an uninterrupted suture, the ends being left long enough to hang outside. The skin was closed by an ordinary interrupted suture, directions being given to pull out the inner suture the third day. These cases were followed by complete recovery.

